# Phenolic Composition, Antioxidant, Anti-Enzymatic, Antimicrobial and Prebiotic Properties of *Prunus spinosa* L. Fruits

**DOI:** 10.3390/foods11203289

**Published:** 2022-10-20

**Authors:** Mirjana Marčetić, Stevan Samardžić, Tijana Ilić, Dragana D. Božić, Bojana Vidović

**Affiliations:** 1 Department of Pharmacognosy, Faculty of Pharmacy, University of Belgrade, 11221 Belgrade, Serbia; 2 Department of Bromatology, Faculty of Pharmacy, University of Belgrade, 11221 Belgrade, Serbia; 3 Department of Microbiology and Immunology, Faculty of Pharmacy, University of Belgrade, 11221 Belgrade, Serbia

**Keywords:** *Prunus spinosa*, blackthorn, phenolic composition, antioxidant activity, enzyme inhibitory activity, antimicrobial properties, prebiotic activity

## Abstract

Blackthorn (*Prunus spinosa* L.) fruit is bluish-black wild fruit traditionally used in nutrition and medicine. It is recently gaining attention as a functional food and an underutilized source of bioactive compounds for application in the food and pharmaceutical industry. This study aimed to assess the health-promoting potential of blackthorn fruits from Serbia by examining their chemical composition and in vitro biological activities. Phytochemical analysis of the blackthorn fruit extracts was performed using LC-DAD-ESI-MS. The total phenolic (TPC), total flavonoid (TFC), total anthocyanin (TAC) content, antioxidant capacity, and enzyme inhibitory activities were determined spectrophotometrically. The antimicrobial and prebiotic properties were tested using the broth microdilution method. Twenty-seven phenolics belonging to the classes of hydroxybenzoic and hydroxycinnamic acids derivatives, flavonoids, and anthocyanins were identified, with caffeoylquinic acid as the most abundant compound. Blackthorn extracts were characterized by notable TPCs, TFCs, and TACs, and free radical scavenging and reducing ability. The enzyme inhibitory effects (IC_50_ = 0.43–2.16 mg/mL) were observed towards *α*-amylase, *α*-glucosidase, acetylcholinesterase, and tyrosinase. Blackthorn fruit extracts in a concentration-dependent manner (0.3–5 mg/mL) stimulated the growth of several probiotic microorganisms and their mixtures, especially the yeast *Saccharomyces boulardii*. Obtained results support further evaluation of the functional food potential of blackthorn fruit.

## 1. Introduction

Blackthorn (*Prunus spinosa* L.) is a thorny shrubby plant species that belongs to the rose family (Rosaceae) and is commonly distributed in most of Europe, Asia Minor, and northwest Africa [1,2]. In late summer and early autumn, it bears bluish-black fruits of astringent taste that are used in diet and traditional medicine. They can be consumed raw, when ripe in autumn, or overripe after the first frosts when fruits become less pungent. Additionally, blackthorn fruits are used to make the compote, jam, brandy, and liqueur [3,4,5].

Ethnopharmacological studies reported that the blackthorn fruit has been used for urinary tract disorders, diabetes, bronchitis, diarrhea, and external inflammatory processes in the mouth and throat [4,6,7]. Previous chemical composition analysis of blackthorn fruit revealed the presence of phenolic constituents, such as hydroxycinnamic acid derivatives, anthocyanins, and flavonol glycosides [8,9,10]. It also contains significant amounts of available carbohydrates and dietary fibers and is characterized by a high content of monounsaturated fatty acids. *Prunus spinosa* fruit can be considered a source of vitamin E [5]. Previous studies demonstrated the antioxidant, antimicrobial and anti-inflammatory properties of blackthorn extracts and their ability to inhibit the growth of different cancer cell lines [10,11,12,13,14]. In fact, many of the health-promoting effects of *P. spinosa* fruits are attributed to polyphenols. Although anthocyanins are recognized as one of the most important bioactive compounds, there is evidence that phenolic acids and flavone/ols, due to synergistic and additive effects, exerted stronger or comparable effects to the anthocyanin-rich fractions of blackthorn fruits [13]. In addition, the alkali-extracted fraction, rich in arabinogalactan and hemicelluloses, exerted radical scavenging activities comparable to vitamin C [15]. Due to seasonality, blackthorn fruits require processing and storage. However, there is evidence that freezing and several months of frozen storage did not significantly influence on nutritive and bioactive compounds of blackthorn fruits [16]. In fact, freeze-drying is the most favorable preservation method compared to convection drying and frozen [17]. However, dried blackthorn fruits, which are characterized by a much lower content of bioactive compounds than fresh fruits, exhibited considerable anti-inflammatory and antioxidant potentials [18]. Therefore, blackthorn fruits are gaining attention not only as “undiscovered” health-promoting food but also as a potential source of pigments, flavors, and bioactive ingredients for functional food and nutraceutical applications [8,19].

Due to the antioxidant and antimicrobial activities of polyphenols, blackthorn fruit extracts could be used as natural colorants and preservatives in the food industry [14,20,21,22]. Adding blackthorn fruits to the ice cream samples improved quality parameters, like color and appearance, gumming structure, and general acceptability [23,24]. Contributing to nutritional value, and functional and sensory properties, blackthorn fruits could be suitable to use as functional additives to food products, such as isotonic beverages, kombucha, probiotic yogurt [25,26,27], and fermented meat products [28]. Cold press oils of *P. spinosa* could have further industrial applications since they are rich in *β*-sitosterol, vanillin, and *γ*-tocopherols [29,30]. In addition, photoprotective effects make blackthorn extracts suitable for usage in cosmetic formulations [31].

There are several reports on the phytochemical composition and antioxidant potential of wild-growing blackthorn fruits from north [10], central [32], and southeast Serbia [33,34,35]. It has been demonstrated that the blackthorn fruit extracts originated from Serbia display in vitro antimicrobial [33,35] and anti-proliferative effects against the HT29 colorectal cancer cell line [10]. Due to the presence of the polyphenolic compounds, blackthorn extracts have been shown the potential to inhibit enzymes related to sugar metabolism (α-amylase and α-glucosidase) [10] and reduce melanin synthesis through the tyrosinase inhibitory effect [32,34]. This study aimed to analyze the phenolic composition and biological activities of blackthorn fruits collected from two localities in central and western Serbia. In addition to antioxidant and enzyme inhibitory activities against α-amylase, α-glucosidase, acetylcholinesterase, and tyrosinase, we assessed the effects of blackthorn fruit extracts on the growth of selected pathogenic and probiotic microorganisms. According to our knowledge, this is the first report on blackthorn fruit’s prebiotic and anti-acetylcholinesterase properties.

## 2. Materials and Methods

### 2.1. Plant Material and Extraction Procedure 

Ripe fruits of *P. spinosa* were collected in September 2020 from two different localities in Serbia: on the slope of mountain Crni Vrh (central Serbia; 44°00′32″ N; 21°06′35″ E) and in the region of Ljig (western Serbia; 44°13′27″ N; 20°14′18″ E) (Supplementary Materials; Figure S1). The plant material was identified by botanist Prof. Dr. Violeta Slavkovska (Department of Botany, University of Belgrade–Faculty of Pharmacy). The voucher specimens were deposited in the herbarium of the Department of Botany (HFF 4278 and HFF 4279), University of Belgrade–Faculty of Pharmacy. Immediately afterward, the fresh plant material was frozen at −20 °C and stored until extraction.

After separating seeds from the epicarp and mesocarp, the fleshy parts of the blackthorn fruits were chopped, intensively rubbed in a mortar, and extracted with methanol, ethanol 50% (*v*/*v*), or water (DER 1:10, *m*/*v*) with constant mixing on a shaker KS 15 A (Edmund Bühler GmbH, Bodelshausen, Germany). After 24 h, macerates were filtered, organic solvents were removed by a vacuum evaporator Rotavapor RII (Büchi Labortechnik AG, Flawil, Switzerland), while the water was removed using a freeze-dryer Alpha 1-4 LDplus (Martin Christ Gefriertrocknungsanlagen GmbH, Osterode am Harz, Germany). Obtained dry blackthorn extracts were stored in a refrigerator at +4 °C until further chemical and bioactivity analysis. Extracts of blackthorn fruits collected in the region of Ljig (western Serbia) were designated as PSE1, whereas extracts of blackthorn fruits collected on the slope of mountain Crni Vrh in central Serbia were designated as PSE2.

### 2.2. Total Phenolic Content

Total phenolic content (TPC) was determined using the *Folin–Ciocalteu* reagent, according to the previously described microassay [36]. The absorbances were measured at 630 nm. A standard of gallic acid with a 10–80 mg/L range was used to construct the calibration curve. The results were expressed as milligrams of gallic acid equivalents per 100 g of fresh fruits (mg GAE/100 g).

### 2.3. Total Flavonoid Content

Total flavonoid content (TFC) was determined by a spectrophotometric method using AlCl_3_, as described by Ordon et al., with slight modification [37]. The absorbances were determined at 490 nm. The calibration curve was constructed using a standard solution of hyperoside (100–800 mg/L) and the results were expressed as milligrams of hyperoside equivalents (HE) per 100 g of fresh fruits (mg HE/100 g).

### 2.4. Total Anthocyanin Content

Total anthocyanin content (TAC) was determined by the pH differential method as described by Oğuz et al., 2019 [38]. The calculations were made according to the following formula:

TAC (mg/100 g) = (A × MW × DF × 10,000)/ε × l (1)

A = absorbance difference, MW = molecular weight (MW: 449.2), DF = dilution factor, ε = molar extinction coefficient (ε: 26,900), l = pathlength (1 cm).

The results were expressed as milligrams of cyanidin 3-glucoside equivalents per 100 g of fresh fruits (mg C3G/100 g).

### 2.5. Phenolic Composition

The HPLC-DAD-ESI-MS technique was employed to examine the phenolic composition of methanol, water, and hydroethanolic extracts of the ripe fruits of *P. spinosa* from two localities in Serbia. The investigation was performed on an Agilent LC-MS system 1260/6130 (Agilent Technologies, Waldbronn, Germany) consisting of a quaternary pump (G1311B), degasser (G1311B), autosampler (G1329B), column compartment (G1316A), DAD detector (G4212B) and a single quadrupole mass detector (6130). Samples were prepared immediately before the analysis by dissolving dry extracts of blackthorn fruits in appropriate solvents (methanol, ethanol 50% (*v*/*v*), water) to obtain a solution with a concentration of 100 mg/mL and subsequent filtering using disposable syringe filters (pore size 0.45 µm, regenerated cellulose membrane, Captiva, Agilent). The injection volume was 5 µL. The separation of the extract components was carried out on a C18 reversed-phase column (Zorbax SB-Aq, 3 × 150 mm, particle diameter 3.5 μm, Agilent Technologies) thermostatted at 25 °C. The mobile phase consisted of component A (0.1% HCOOH) and component B (acetonitrile). The elution program was optimized to achieve the best possible separation of the extract constituents and was as follows: 0–40 min (5–25% B), 40–44 min (25–90% B), 44–45 min (90–90% B), 45–50 min (90–5% B). The mobile phase flow rate was constant (0.35 mL/min). UV spectra were generated in the range 190–640 nm and UV chromatograms were recorded at 210, 280, 320, 350 and 520 nm. Analytes fragments were produced using electrospray. The parameters of the spray chamber were as follows: gas temperature (350 °C), drying gas flow (10 L/min), nebulizer pressure (40 psi), and capillary voltage (3500 V). The fragmentation voltages used were 100, 200 and 250 V, which enabled information on the molecular mass and structure of the analytes. Mass spectra were acquired in the *m/z* range 100–1200 in the negative (phenolic acids and flavonoids) and positive polarity (anthocyanins). The generated data were processed using ChemStation software Rev. B.04.03 SP1. The identification was performed in comparison with standard compounds or tentatively by comparing the experimentally generated spectral data with the corresponding literature data [8,10,13,39,40,41]. The content of the major compound (caffeoylquinic acid 1) was determined at 320 nm and expressed as milligrams of chlorogenic acid equivalents (CAE) per 100 g of extract (mg CAE/100 g), whereas the content of the most abundant flavonoid quercetin pentoside 3 was determined at 350 nm and expressed as milligrams of quercetin equivalents (QE) per 100 g of extract (mg QE/100 g). The external standard method was used for quantification (chlorogenic acid calibration curve: y = 25,999x − 12.0567, R^2^ = 0.99996, concentration range (c) = 0.00183–1.10000 mg/mL, LOD = 0.00999 mg/mL, LOQ = 0.03027 mg/mL; quercetin calibration curve: y = 42228x + 2.7938, R^2^ = 0.99999, c = 0.00080–0.12000 mg/mL, LOD = 0.00070 mg/mL, LOQ = 0.00211 mg/mL).

### 2.6. Antioxidant Activities of Blackthorn Extracts

Four in vitro assays were used to evaluate the antioxidant activities of blackthorn extracts. Radical scavenging activities were tested by 2,2′-diphenyl-1-picrylhydrazyl (DPPH) and 2,2′-azino-bis(3-ethylbenzothiazoline)-6-sulphonic acid (ABTS) assays, reducing power by ferric reducing antioxidant power (FRAP) assay, while lipid peroxidation ability was analyzed by *β*-carotene bleaching inhibition assay. All assays were performed using 96-well microplates and a microplate reader ELx800 Absorbance Microplate Reader (BIOTEK, Santa Clara, CA, USA).

#### 2.6.1. DPPH Assay

The radical scavenging ability of blackthorn extracts against DPPH radical was investigated using a microassay described by Melendez et al. [42]. The absorbances were measured at 490 nm. The calibration curve was constructed by Trolox (0.2–0.7 mM). The results were expressed as mM of Trolox equivalents per 100 g of fresh fruits (mM TE/100 g).

#### 2.6.2. ABTS Assay

Radical scavenging activity against ABTS radicals was evaluated using a previously described method [43]. The absorbances were measured at 630 nm. A standard of Trolox with a 0.2–1.5 mM range was used to construct the calibration curve. The results were expressed as mM of Trolox equivalents per 100 g of fresh fruits (mM TE/100 g).

#### 2.6.3. FRAP Assay

The ability of blackthorn extracts to reduce the ferric tripyridyltriazine complex to the ferrous tripyridyltriazine was evaluated according to Bolanos de la Torre et al. (2015), with some modifications [44]. The absorbances were measured at 630 nm. A standard of Trolox with a 0.1–0.8 mM range was used to construct the calibration curve. The results were expressed as mM of Trolox equivalents per 100 g of fresh fruits (mM TE/100 g).

#### 2.6.4. β-Carotene Bleaching Inhibition Assay

The capacity of blackthorn extracts to reduce oxidative degradation of *β*-carotene was evaluated according to Reis et al. [45]. Sample and *β*-carotene/linoleic acid emulsion were mixed and absorbances of the mixture were measured immediately (t = 0 min) and after the incubation period (t = 120 min) at 490 nm. The results were expressed as a percentage of *β*-carotene oxidative degradation inhibition.

#### 2.6.5. Antioxidant Composite Index

Based on the obtained results using DPPH, ABTS, FRAP, and *β*-carotene bleaching inhibition tests, an overall antioxidant composite index (ACI) for blackthorn fruit extracts was calculated. For each assay, an index value of 100 was assigned to the best score. The mean value of all four assays presented the ACI value for analyzed samples [46].

### 2.7. In Vitro Enzyme Inhibitory Activities of Blackthorn Extracts

#### 2.7.1. α-Amylase Inhibitory Activity Assay

The modified method reported by Ahmed et al. was used to evaluate the α-amylase inhibitory activity of methanol blackthorn extracts [47].

Serial dilutions of the samples and enzyme (*α*-amylase type VI-B, ≥10 units/mg solid, Sigma- Aldrich, St. Louis, MO, USA) in a phosphate buffer (pH 6.9) were incubated at 37 °C for 15 min. Then, 1.0% starch solution was added, and 3,5-dinitro salicylic acid color reagent solution. The absorbances were measured at 540 nm. The percentage inhibition was calculated using the formula:

% Inhibition = [(Ac − As)/Ac] × 100,(2)

where Ac is the absorbance of the control (without sample) and As is the absorbance of the samples at different concentrations. The results expressed as IC_50_ values (mean ± standard deviation) were obtained by linear regression from curves describing the dependence of inhibition (%) on sample concentration (mg/mL). Acarbose was used as the reference standard.

#### 2.7.2. α-Glucosidase Inhibitory Activity Assay

Inhibitory activities of methanol blackthorn extracts against *α*-glucosidase were tested according to the modified method reported by Indrianingsih et al. [48]. Serial dilutions of the samples, *p*-nitrophenyl-*α*-D-glucopyranoside solution, and phosphate buffer (pH 5.0) were mixed and incubated at 37 °C for 10 min. Then, the enzyme (*α*-glucosidase type I, ≥10 units/mg solid, Sigma-Aldrich, St. Louis, MO, USA ) was added and incubated for 30 min. The reaction was stopped by adding 0.2 M Na_2_CO_3_, and the absorbance was measured at 405 nm. The percentage inhibition was calculated using the formula:

% Inhibition = [(Ac − As)/Ac] × 100,(3)

where Ac is the absorbance of the control (without sample) and As is the absorbance of the samples. The results expressed as IC_50_ values were obtained by linear regression from curves describing the dependence of inhibition (%) on sample concentration (mg/mL). Acarbose was used as the reference standard.

#### 2.7.3. Acetylcholinesterase Inhibitory Activity Assay

The ability of methanol blackthorn extracts to inhibit acetylcholinesterase was evaluated following the method reported by Ellman et al. with slight modification [49].

Serial dilutions of the samples, enzyme (acetylcholinesterase type VI-S, 222 units/mg solid, Sigma-Aldrich, St. Louis, MO, USA), and Tris buffer (pH 8.0) were mixed and incubated at 37 °C for 15 min. The reaction was initiated by adding acetylcholine iodide and 5,5′-dithiobis-2-nitrobenzoic acid. After incubation for 30 min at 37 °C, the absorbance was measured at 412 nm. The percentage inhibition was calculated using the formula:

% Inhibition = [(Ac − As)/Ac] × 100,(4)

where Ac is the absorbance of the control (without sample) and As is the absorbance of the samples. The results expressed as IC_50_ values were obtained by linear regression from curves describing the dependence of inhibition (%) on sample concentration (mg/mL). Galantamine was used as the reference standard.

#### 2.7.4. Tyrosinase Inhibitory Activity Assay

The assessment of tyrosinase inhibitory activities of methanol blackthorn extracts was performed according to the modified method described by No et al. [50]. Serial dilutions of the samples, enzyme (tyrosinase, ≥1000 units/mg solid, Sigma-Aldrich, St. Louis, MO, USA), and phosphate buffer (pH 6.6) were mixed and incubated at 30 °C for 10 min. The absorbance was measured at 490 nm after adding L-tyrosine and incubating for 30 min at 30 °C. The percentage inhibition was calculated using the formula:

% Inhibition = [(Ac − As)/Ac] × 100, (5)

where Ac is the absorbance of the control (without sample) and As is the absorbance of the samples. The results expressed as IC_50_ values were obtained by linear regression from curves describing the dependence of inhibition (%) on sample concentration (mg/mL). Kojic acid was used as the reference standard.

### 2.8. Antimicrobial Activity and Prebiotic Potential of Blackthorn Extracts

#### 2.8.1. Bacterial/Yeast Strains and Culture Media

The antimicrobial activity of methanol blackthorn extracts was tested against laboratory control strains of eight pathogenic bacteria and one yeast derived from ATCC and NCIMB collection (KWIK-STIK™, Microbiologics, St. Cloud, MN, USA). The prebiotic activity of blackthorn extracts in terms of growth stimulation potential was evaluated using six probiotic strains: *Lactobacillus* spp. (3 strains), *Streptococcus salivarius* subsp. *thermophilus* (1 strain) and *Saccharomyces* *boulardii* (2 strains), and two mixtures of lactobacilli/bifidobacterium strains with or without *S. boulardii* (Supplementary Materials; Table S1). Strains were maintained at −80 °C in 15% (*w*/*w*) glycerol, defrosted before experiments, and inoculated from stock solutions onto Triptic Soy agar/broth (TSA/TSB, Oxoid Ltd., Basingstoke, Hampshire, UK) for bacteria, Sabouraud Dextrose agar/broth (SDA/SDB, Oxoid) for *Candida albicans* and *Saccharomyces boulardii* strains, and *Lactobacillus* agar/broth according to de Man Rogosa and Sharpe (MRSa/MRSb) (Oxoid) for probiotic bacterial strains. Before experiments, all tested strains were incubated on corresponding agar plates for 24–48 h at 35 °C under aerobic or microaerophilic conditions (for lactobacilli), and fresh (overnight) cultures were used for the preparation of inoculum.

#### 2.8.2. Antimicrobial Activity

The antimicrobial activity of blackthorn methanol extracts was determined by broth microdilution test in 96-well microtiter plates according to the European Committee on Antimicrobial Susceptibility Testing guidelines [51]. The suspensions of microorganisms were made in a saline solution to a density of 0.5 per McFarland standard (Bio-Merieux Craponne, France). Extracts were dissolved in DMSO, and further prepared in concentrations ranging from 0.15–5.00 mg/mL in fresh Mueller-Hinton broth (MHB, Lab M Limited, Lancashire, UK) for bacteria, SDB for yeasts, and MRSb for lactobacilli. Each concentration was set in duplicate and inoculated with 5 × 10^5^ CFU/mL of microorganisms. To detect cell growth and metabolism, MHB/SDB/MRSb were supplemented with 0.05% 2,3,5-triphenyltetrazolium chloride (TTC, Sigma-Aldrich, St. Louis, MO, USA). TTC is a redox indicator and a colorless dye that becomes a red metabolite 1,3,5-triphenylformazan due to the activity of cellular dehydrogenase and is used to differentiate alive from dead cells. Minimal inhibitory concentrations (MIC) were determined after incubation for 20–48 h at 35 °C in aerobic/microaerophilic conditions as the lowest concentration of extract that inhibits growth (i.e., shows no visible change of medium color). Positive controls (microorganisms in medium) and negative controls (only medium with extracts) were included in the experiments. Each test was repeated three times.

#### 2.8.3. Prebiotic Activity

The suspensions of probiotic microorganisms (Supplementary Materials; Table S1) were made in a saline solution to a density of 0.5 per McFarland standard (Bio-Merieux). Blackthorn methanol extracts were dissolved in DMSO, and further prepared in concentrations ranging from 0.31–5.00 mg/mL in fresh TSB, SDB, and MRSb. Each concentration was set in triplicate and inoculated with 5 × 10^5^ CFU/mL of microorganisms. Positive controls (microorganisms in medium) and negative controls (only medium with extracts) were set in triplicates. Plates were incubated for 20–48 h at 35 °C in aerobic/microaerophilic conditions, and optical density was measured at 600 nm for bacteria and 530 nm for yeasts using a microtiter plate reader (Multiskan™ FC Microplate Photometer, ThermoFisher Scientific, Waltham, MA, USA). Each test was repeated three times.

The prebiotic activity of blackthorn extracts was calculated as a mean value of nine total measurements for each strain/concentration of extract and expressed as a percent of growth, compared to the growth of positive controls (expressed as 100%) with the formula:

% of prebiotic effect = OD_sample_ × 100/OD_positive control_. (6)

### 2.9. Statistical Analysis

All analyses were done at least in triplicate, and the results were expressed as the mean values ± the standard deviations (SD). Statistical analysis was performed by the one-way analysis of variance (ANOVA) with Tukey HSD test, independent samples t-test, and Pearson correlation by using SPSS software (version 20, Chicago, IL, USA), and *p* values < 0.05 were considered statistically significant.

## 3. Results and Discussion

### 3.1. Total Phenolic, Total Flavonoid and Total Anthocyanin Contents

The total phenolic content (TPC) of blackthorn fruit extracts ranged from 152.22 to 321.36 mg GAE/100 g (Table 1). Regardless of the localities of collection, methanol extracts were the richest in phenols, followed by hydroethanolic extracts, while water extracts were the poorest in phenols. Differences in TPCs in methanol and hydroethanolic extracts were insignificant (*p* > 0.05). However, TPCs in water extracts were significantly lower than in methanol and hydroethanolic extracts (*p* < 0.05). All extracts of blackthorn fruit collected from the location in western Serbia (PSE1) had significantly higher levels of TPC than the corresponding extracts of blackthorn fruit from the location in central Serbia (PSE2).

The total flavonoid content (TFC) of analyzed blackthorn fruit extracts ranged from 28.18 to 67.88 mg HE/100 g (Table 1), with the highest content observed in hydroethanolic extracts. The significantly higher TFCs were in PSE1 than in PSE2 extracts (*p* < 0.05).

Total anthocyanin content (TAC) ranged from 0.68 to 14.38 mg C3G/100 g (Table 1). Regarding these specialized metabolites, the highest content was found in methanol, followed by hydroethanolic, and the lowest TAC was found in aqueous extracts. In contrast to TPC and TFC, the higher TAC was determined in methanol and hydroethanolic PSE2 extracts.

According to previous investigations, the content of TPC in methanol extract of blackthorn fruits from central Serbia was 11.24–18.70 mg GAE/g [32] and 11.10–30.43 mg GAE/g in fruits from northern Serbia [10]. Additionally, significant TFC (5.32–46.08 mg/100 g) and TAC (35.70–423.30 mg/100 g) content was reported in these samples [10]. The water, ethanol, and acetone extracts of fruits from Croatia contained marked amounts of TPC (19.98–26.78 mg GAE/g) and TFC (2.89–3.07 mg QE/g) [52]. Lower amounts of TPC and TFC in our samples could be due to different genetic and environmental factors as well as the processing method that could lead to the decomposition of phenolic compounds.

### 3.2. Phenolic Composition

Phytochemical analysis of the methanol, ethanol 50% (*v*/*v*), and aqueous blackthorn fruit extracts revealed the presence of twenty-seven phenolics (Supplementary Materials; Table S2), belonging to the different classes of specialized metabolites, i.e., hydroxybenzoic acid derivatives, hydroxycinnamic acid derivatives, flavonoids, and anthocyanins. The only representative of hydroxybenzoic acid derivatives was vanillic acid hexoside, whereas hydroxycinnamic acid derivatives included caffeoylquinic acids, feruloylquinic acid, and caffeoylshikimic acid. Flavonoids were mainly present in the form of various glycosides of quercetin, methylquercetin, and kaempferol, and the presence of aglycone quercetin was also confirmed. Anthocyanins were detected in the forms of complexes of cyanidin and peonidin with a monosaccharide or disaccharide unit. Based on the peak area of the recorded chromatograms, the dominant compound was caffeoylquinic acid 1 (83.00–836.09 mg CAE/100 g). Among flavonoids, the highest content of quercetin pentoside 3 was noticed (6.43–54.56 mg QE/100 g).

The phytochemical composition of the studied *P. spinosa* extracts follows the available literature data. Except for methylquercetin pentosides and kaempferol deoxyhexoside, the presence of all other phenolics has been reported before [8,10,13]. However, these phenols are constituents of other parts of *P. spinosa* or other species of the genus *Prunus*. Kaempferol 3-*O*-α-L-rhamnopyranoside (afzelin) and kaempferol 7-*O*-α-L-ramnopyranoside were detected in the blackthorn flowers [40], whereas isorhamnetin 3-*O*-α-arabinofuranoside and isorhamnetin 3-*O-*β-xylopyranoside are flavonoids whose presence was proven in the *P. serotina* leaves [41]. Quantitative analysis data (Table 2) are also consistent with previous findings. The most abundant classes of compounds in the blackthorn fruits are hydroxycinnamic acids and anthocyanins [8,10,13]. In the lyophilized fruits from northern Serbia, hydroxycinnamic acids: 3-*O*-caffeoylquinic acid (neochlorogenic acid), 5-*O*-caffeoylquinic acid (chlorogenic acid), 3-*p*-coumaroylquinic acid and anthocyanins: cyanidin 3-glucoside, cyanidin 3-rutinoside, peonidin 3-glucoside and peonidin 3-rutinoside were major constituents. Flavonoids: quercetin 3-galactoside, quercetin 3-glucoside, quercetin 3-rutinoside and quercetin aglycone were also present. The concentrations of 3-*O*-caffeoylquinic acid (46.63–636.14 mg/100 g dw), 5-*O*-caffeoylquinic acid (1.70–30.92 mg/100 g dw) were in the same range as our results (caffeoylquinic acid 1, 83.00–836.09 mg/100 g). Previously investigated blackthorn fruit from northern Serbia contained significant amounts of anthocyanins cyanidin 3-glucoside (4.41–183.63 mg/100 g dw) and cyanidin 3-rutinoside (7.38–185.62 mg/100 g dw) [10]. The main phenolic compound in the fruits originated from central Serbia was quercetin 3-rutinoside (3.39–5.90 mg/100 g), followed by kaempferol 3-glucoside (0.62–0.86 mg/100 g), quercetin 3-galactoside, naringin and aesculin [32]. In line with obtained results, the most abundant constituent in hydromethanolic extract of blackthorn fruits collected in Northeastern Portugal and Poland was 3-*O*-caffeoylquinic acid (22.09 and 15.56 mg/100 g dw, respectively) [8,13].

Although comparative analysis of phytochemical fingerprints indicated a qualitative similarity of the tested extracts, there were noticeable differences among them in the content of dominant compounds. In the PSE1 extracts, the caffeoylquinic acid 1 content ranged from 482.09 to 836.09 mg CAE/100 g. The highest content was determined in the methanol extract and the lowest in the aqueous extract. The hydroethanolic extract was moderately rich and contained 776.50 mg CAE/100 g. The same pattern was observed for the dominant flavonoid quercetin pentoside 3 content. Namely, this compound was the most abundant in the methanol extract (54.56 mg QE/100 g), the lowest content was found in the aqueous extract (19.77 mg QE/100 g), whereas the medium content was present in the hydroethanolic extract (32.85 mg QE/100 g). In general, it was evident that the PSE2 extracts had less phenolics. In these extracts, caffeoylquinic acid 1 and quercetin pentoside 3 contents were 83.00–559.39 mg CAE/100 g and 6.43–25.47 mg QE/100 g, respectively. For the PSE2, the methanol extract had higher contents of these phenols than hydroethanolic and water extracts. It was previously reported that 3-caffeoylquinic acid contents in hydromethanolic extracts of blackthorn fruits originating from Poland were 452 and 1556 mg/100 g [13,18]. Although a comparison with our results is not possible due to the differently expressed results, 3-caffeoylquinic acid contents varied in dried blackthorn fruits in northern Serbia, from 46.63 to 636.14 mg/100 g, depending on the location and genotype [10].

In general, our results indicate the variability in the chemical composition of extracts, which depends on the extraction solvent used and the localities of blackthorn fruits. Considering that environmental factors influence biosynthesis and accumulation of secondary metabolites, observed differences in the content of phenolic compounds between the same type of extracts from blackthorn fruits from different locations could be explained by differences in the altitudes, insolation, temperature, rainfall precipitations, and soil characteristics [8,53]. Due to the highest total phenolic and total anthocyanin contents, methanol extracts of blackthorn fruits were used for further evaluation of bioactivity.

### 3.3. Antioxidant Activities

Antioxidant potentials of the methanol *P. spinosa* fruit extracts from the location in western Serbia (PSM1), and the location in central Serbia (PSM2) were evaluated using different in vitro assays. Based on the results summarized in Table 3, it could be noticed that blackthorn extracts from different localities exerted statistically significant differences in antioxidant properties. In particular, the PSM1 extract demonstrated higher antioxidant activity in DPPH, ABTS, and FRAP assays than the PSM2. Based on the ACI value, the PSM1 extract had about 1.3-fold higher antioxidant capacity than the PSM2. These results may be related to the fact that the PSM1 extract had a significantly higher content of total phenolic compounds. In agreement with previous findings [9,10,52,54], strong positive correlations were found among the antioxidant activity in different assays and the total phenolic content (Supplementary Materials; Table S3). On the other hand, we did not observe any significant correlations between antioxidant activity and TFC, while the TAC showed a strong linear correlation only with the *β*-carotene bleaching assay. However, the absence of a significant difference between *P. spinosa* extracts for this assay indicates that besides anthocyanins, other compounds contribute to inhibiting lipid peroxidation abilities. In addition to phenolic compounds, other phytochemicals, such as organic acids, vitamins (ascorbic acid, tocopherols), and carotenoids, could be related to the antioxidant properties of blackthorn fruits [55,56].

### 3.4. Enzymatic Inhibitory Effects

Inhibition of α-amylase and α-glucosidase may decrease the rate of glucose absorption into the blood and suppress postprandial hyperglycemia. Therefore, using plant extracts as carbohydrate digestive enzyme inhibitors may facilitate the processes of glucose homeostasis [57]. It has already been found that extracts from different *Prunus* fruits, including blackthorn, have anti-diabetic properties [9,57]. Previous studies reported that both the genotype and the type of solvent used for the extraction significantly affected blackthorn in vitro inhibition of carbohydrates hydrolyzing enzymes [10,52]. Veličković et al. showed that the hydroethanolic *P. spinosa* extract displayed a significantly stronger ability to inhibit *α*-amylase and *α*-glucosidase than the water extract, which may be due to its high flavonoid content [52]. It was also demonstrated that among 15 wild-growing blackthorn genotypes from northern Serbia, genotypes with higher anthocyanin content possessed higher activity against α-amylase, while α-glucosidase inhibitory activity positively correlated with quercetin glucosides content [10]. Recent in silico analysis pointed out that among phenol compounds in different *Prunus* fruits, the most potent inhibitor of α-amylase is cyanidin 3-*O*-rutinoside. In contrast, quercetin 3-*O*-rutinoside and cyanidin 3-*O*-(2′-xylosyl)-rutinoside are the most effective inhibitors of α-glucosidase [9].

Our findings demonstrate that methanol *P. spinosa* extracts exhibited both α-amylase and α-glucosidase inhibitory activities. The IC_50_ values for analyzed extracts and tested controls are presented in Table 3. Despite considerable variability in enzyme inhibition abilities of blackthorns from different localities, both samples were more potent inhibitors of α-glucosidase than α-amylase. Previous studies reported similar results [10,52]. Our data showed that the PSM2 extract was a more efficient α-amylase inhibitor than the PSM1 extract, possibly due to higher anthocyanins content (Table 1 and Table 3).

Apart from catalyzing the hydrolysis of the neurotransmitter acetylcholine, the enzyme acetylcholinesterase (AChE) has many different functions, including the modulation of oxidative stress, inflammatory and apoptotic response, as well as morphogenic and adhesion functions. In addition to antioxidant and anti-inflammatory properties, there is evidence that bioactive compounds from various *Prunus* species exert neuroprotective effects by AChE inhibition [58]. However, to our best knowledge, there are no previous data regarding the AChE inhibitory capacity of *P. spinosa* fruit extracts. We have observed that the PSM1 extract, characterized by the higher contents of phenolic acids and flavonoids, showed about 4-fold higher AChE inhibitory activity than the PSM2 extract. However, both extracts presented lower inhibitions of AChE compared to the positive control, galantamine (Table 3).

Tyrosinase is an enzyme involved in the oxidation of tyrosine to melanin which overproduction is associated with hyperpigmentation, photo-carcinogenesis of skin, and neurodegenerative processes [59]. Previous data on the tyrosinase inhibitory activity of *P. spinosa* fruit are very limited. Stanković et al. have observed considerable tyrosinase inhibition ability for the 45% (*v*/*v*) propylene glycol extract, suggesting that it could be ascribed to its higher total phenolics and antioxidant activity than those in other extracts (water, 70 % (*v*/*v*) ethanol, and methanol) [34]. Other authors proposed that the presence of ellagic and vanillic acids and the metal chelation ability could be important factors determining the antityrosinase properties of blackthorn fruit extracts [32]. In contrast, we demonstrated that extract (PSM2) with lower total phenolics and less reducing and overall antioxidant capacity had higher antityrosinase activity. However, both extracts exerted lower activities than kojic acid (Table 3).

### 3.5. Antimicrobial Activity

The pathogen strains of Gram-positive and Gram-negative bacteria and yeast were purposely selected to represent the target microorganisms that could be inhibited after oral consumption of blackthorn as functional foods or food supplements. They included the representatives of common human microbiota, enteric and foodborne pathogens. Antimicrobial activity was also tested against probiotic microorganisms to determine if there was any inhibition of their growth and to define concentrations for evaluating of the prebiotic activity of blackthorn extracts.

Various plant extracts are usually more effective against Gram-positive than Gram-negative bacteria due to the different structures of their outer layer [60]. Gram-positive bacteria have a thick porous cross-linked polymer peptidoglycan in their cell wall. Gram-negative bacteria have a more complex structure with two cell membranes that interfere with the transport of nutrients and other molecules through the wall. Our results showed modest antimicrobial activity of blackthorn extracts against pathogenic bacteria and yeasts, with MIC values of 1.25–5.00 mg/mL for *S. aureus*, *S. epidermidis*, *E. coli*, *K. pneumoniae*, *S. abony* and *P. aeruginosa* (Table 4). The PSM1 had better inhibitory activity and lower MIC values than the PSM2. Although moderate, antimicrobial activity of both extracts against *Escherichia* and *Salmonella* is a significant finding since these bacteria represent enteric pathogens that cause severe diarrheal syndromes, like travelers’ diarrhea or gastroenteritis (salmonellosis). Other pathogenic and probiotic microorganisms were not inhibited by blackthorn extracts (MIC > 5 mg/mL).

Our results are in accordance with previous research that showed inhibitory activity of blackthorn ethanol extract on the growth of Gram-positive and Gram-negative bacteria in concentrations from 4.36 to 8.72 mg/mL [53], 10 to 30 mg/mL [33] and 5.68 to 11.36 mg/mL [52]. Extract PSM1 inhibited *E. coli* in the concentration of 2.5 mg/mL and *S. abony* in the concentration of 5 mg/mL, which is almost twofold lower compared to the results of Sabatini et al. (4.36 mg/mL and 8.72 mg/mL, respectively) [53] and Veličković et al. (for both strains 11.36 mg/mL for water extract and 5.68 mg/mL for ethanol blackthorn extract) [52]. On the other hand, Radovanović et al. [35] reported the antibacterial activity of the phenol-rich extracts obtained from blackthorn fruits grown in southeastern Serbia against Gram-positive and Gram-negative bacteria, with significantly lower MIC values (0.0156–0.5 mg/mL) compared to ours, and results of other authors, probably due to a very high content of total phenols (795.99 mg/100 g vs. 152.22–321.36 mg/100 g determined in this study).

The possible mechanisms of antimicrobial activity of plant extracts have been extensively studied. They include the damage to the bacterial cell membrane, suppression of virulence factors (toxins), alteration/inactivation of essential cellular enzymes, disruption of microbial membranes, loss of macromolecules, and cell death [61,62]. Previous studies have shown that flavonoids and phenolic acids in blackthorn extracts could be responsible for antimicrobial activity [14]. Therefore, considering that our extracts were rich in phenolics, it seems highly possible that these compounds contributed to the observed antimicrobial effects.

In previous studies, hydroxycinnamic acid derivatives (3-caffeoylquinic, 4-caffeoylquinic and 5-caffeoylquinic acids) exhibited antibacterial and antifungal activity against several microorganisms, i.e., *Staphylococcus aureus*, *Escherichia coli*, *Salmonella enterica*, *Vibrio parahaemolyticus*, *Candida albicans*, *Saccharomyces cerevisiae* and *Aspergillus niger* [63]. Considering these findings and the fact that in PSM1 and PSM2, the quantitatively dominant compound was caffeoylquinic acid, there is a high possibility that this compound is mainly responsible for the demonstrated antimicrobial effects. The contribution of flavonoids and anthocyanins is also possible [64,65]; however, their concentrations in the examined extracts were significantly lower.

### 3.6. Prebiotic Potential

The prebiotic activity of various red fruit extracts and plant extracts rich in phenols has been previously studied [62,66]. However, there is no evidence of the effects of blackthorn extracts on the growth of beneficial microorganisms. Therefore, we decided to investigate the prebiotic activity of blackthorn fruit extracts on various strains of probiotic microorganisms commonly used to prevent antibiotic-associated diarrhea and intestinal dysbiosis.

Several studies reported the stimulatory effect of subinhibitory concentrations of sour cherry (*Prunus cerasus* L.) [67], blueberry (*Vaccinium corymbosum* L.) [68], or blackthorn extracts [53] on the growth of various microorganisms, that could be designated as a prebiotic-like effect. Hence, we investigated the potential prebiotic activity of blackthorn methanol extracts in two-fold serial dilutions in the concentration ranges that did not inhibit the growth of probiotic strains in the previous antimicrobial activity testing. The prebiotic activity was calculated compared to the growth of strains without adding blackthorn extracts. 

**Figure 1:**
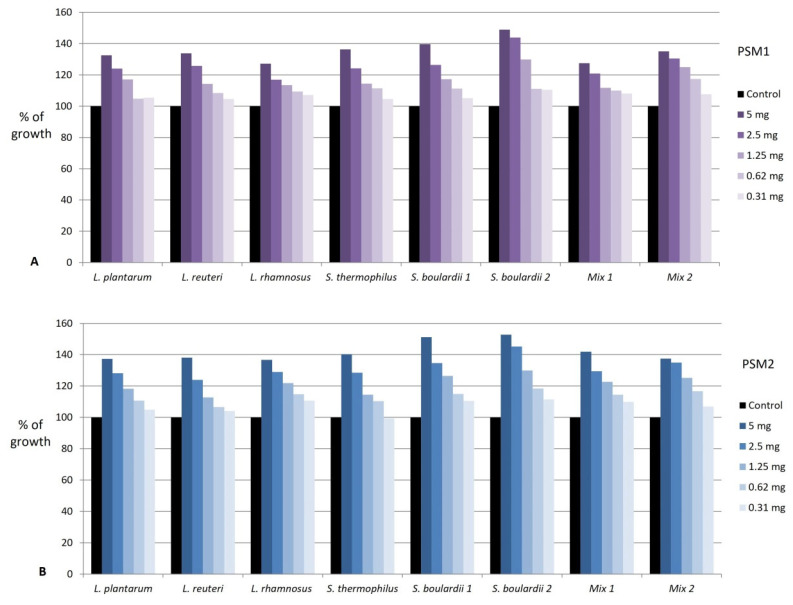
The prebiotic activity of blackthorn extract PSM1 (**A**) and PSM2 (**B**) on various probiotic strains of microorganisms. PSM1—methanol extract of *P. spinosa* fruit from the location in western Serbia; PSM2—methanol extract of *P. spinosa* fruit from the location in central Serbia; Results are presented as a percent of growth compared to the positive control (100% of growth). Mix 1: *L. helveticus*, *L. rhamnosus*, *B. longum*; Mix 2: *L. helveticus*, *L. rhamnosus*, *B. longum*, *S. boulardii*.

Both methanol PSM1 and PSM2 extracts exhibited pronounced prebiotic effects on all probiotic strains in concentrations ranging from 0.3-5.0 mg/mL, with no marked difference between them (Figure 1). The prebiotic activity depended on the type of probiotic strain and concentration of extracts and was in the range of 104.0–152.8% of growth (Supplementary Materials; Table S4) (Figure 1). Stimulation of growth of all probiotic strains was highest in the concentration of 5 mg/mL of PSM1 and PSM2 (135.1 ± 7.0% and 142.1 ± 6.4%, respectively). It slowly decreased to less than 110% of the growth in the concentration of 0.31 mg/mL of PSM1 and PSM2 (106.6 ± 2.1% and 107.2 ± 4.2%, respectively). Among all tested probiotics, the greatest growth stimulation was achieved in both strains of yeast *S. boulardii* in the concentration of 5 mg/mL of blackthorn extracts.

The results of our study revealed that blackthorn extracts possess strong prebiotic activity when applied in concentrations that could be achieved after ingestion of blackthorn fruits or their supplements. Although the literature on *Prunus* prebiotic activity is not available, our results can be compared to other investigations on the prebiotic activity of plant extracts rich in phenols. Our results are in accordance with Milutinović et al., who reported prebiotic activities of various plant extracts rich in phenols on several probiotic strains, with the most pronounced effect on probiotic yeast *Saccharomyces boulardii* [62]. Conversely to our results that both blackthorns extracts stimulated the growth of *L. rhamnosus*, the growth of two *L. rhamnosus* strains investigated in their study was not stimulated by the addition of any of the five plant extracts in the tested concentrations (0.25–10 mg/mL). Sabattini et al. also demonstrated that *P. spinosa* extract in concentrations ranging from 1.15 to 9.13 mg/mL exerted the highest stimulatory activity on microbial growth. However, these authors investigated the effect on various human pathogens and not probiotic strains of microorganisms [53].

The observed prebiotic effects of blackthorn extracts in this study could be attributed to the presence of various bioactive compounds. For example, Wang et al. have recently summarized articles on the effects of anthocyanins and concluded that these compounds could inhibit the growth of pathogenic bacteria through the downregulation of gene expression, alteration of metabolic enzymes, and disruption of respiratory metabolism. However, anthocyanins could also stimulate the growth of probiotics by increasing the production of short-chain fatty acids, and stimulating enzyme activities essential for their proliferation [69]. In addition, there is evidence that a highly purified mixture of three dicaffeoylquinic acids (3,4-, 3,5- and 4,5-dicaffeoylquinic acid) which are similar to the main component of the blackthorn extracts (caffeoylquinic acid) in this study, increased the relative abundances of *Bifidobacterium* and *Akkermansia* in experimental animals [70]. However, prebiotic effects could also be attributed to phenol metabolites. Namely, it has been shown that buccal and gastric digestions have no substantial effects on phenolic compounds of *P. spinosa*. However, these compounds were transformed through intestinal digestion into breakdown products with potential bacteria-stimulatory effects [54].

## 4. Conclusions

The polyphenolic composition analysis revealed the presence of 27 phenolics belonging to the classes of hydroxybenzoic acid derivatives, hydroxycinnamic acid derivatives, flavonoids, and anthocyanins, including methylquercetin pentosides and kaempferol deoxyhexoside, reported for the first time in blackthorn fruits. The dominant compound was caffeoylquinic acid, and among flavonoids, the most abundant was quercetin pentoside. In vitro studies confirmed the antioxidant and enzyme inhibitorypotential of blackthorn fruits. In this work, we first reported the AChE inhibitory effects of blackthorn fruit extracts. However, in contrast to the AChE inhibitory activity, obtained results suggest that phenolics and antioxidant capacity are probably not the primary factors determining the antitirosynase properties of blackthorn fruit extracts. In addition to the modest antimicrobial activity against pathogenic bacteria and yeasts, for the first time, we provided evidence of the potential prebiotic potential of blackthorn fruits, especially on the growth of the *Saccharomyces boulardii*. In conclusion, this work supports the functional food properties of the fruit of *P. spinosa* or its preparations and further investigations to fully exploit the benefits of this widespread wild fruit.

## Figures and Tables

**Table 1:** Total phenolic (TPC), total flavonoid (TFC) and total anthocyanin (TAC) contents of blackthorn fruit extracts.

Parameter	Solvent	PSE1	PSE2
TPC (mg GAE/100 g)	Water	164.57 ± 3.85 ^Ab^	152.22 ± 4.90 ^Bb^
	Methanol	321.36 ± 9.13 ^Aa^	217.04 ± 17.99 ^Ba^
	Ethanol 50% (*v*/*v*)	318.27 ± 14.85 ^Aa^	212.72 ± 13.90 ^Ba^
TFC (mg HE/100 g)	Water	46.06 ± 0.52 ^Ab^	34.24 ± 1.39 ^Bab^
	Methanol	37.58 ± 5.91^Ab^	28.18 ± 2.73 ^Ab^
	Ethanol 50% (*v*/*v*)	67.88 ± 1.05 ^Aa^	39.70 ± 3.19 ^Ba^
TAC (mg C3G/100 g)	Water	2.22 ± 0.03 ^Ac^	0.68 ± 0.02 ^Bc^
	Methanol	9.47 ± 0.21 ^Ba^	14.38 ± 0.95 ^Aa^
	Ethanol 50% (*v*/*v*)	4.11 ± 0.05 ^Bb^	7.86 ± 0.04 ^Ab^

PSE1—extracts of *P. spinosa* fruit from the location in western Serbia; PSE2—extracts of *P. spinosa* fruit from the location in central Serbia; TPC—total phenolic content; TFC—total flavonoid content; TAC—total anthocyanin content; GAE—gallic acid equivalents; HE—hyperoside equivalents; C3G—cyanidin 3-glucoside equivalents; Different letters within the same row ^(A,B)^ and column ^(a–c)^ indicate significant differences (*p* < 0.05).

**Table 2:** Contents (mg/100 g) of phenols in blackthorn fruit extracts.

Compound	PSE1			PSE2		
	Methanol	Water	Ethanol 50%	Methanol	Water	Ethanol 50%
Vanillic acid hex	nq	nq	nq	nq	nq	nq
**Caffeoylquinic acid 1**	836.09 ± 0.37 ^Aa^	482.09 ± 7.41 ^Ac^	776.50 ± 1.32 ^Ab^	559.39 ± 0.62 ^Ba^	83.00 ± 0.42 ^Bc^	402.76 ± 1.16 ^Bb^
Feruloylquinic acid	35.1 ± 0.05 ^Ab^	42.32 ± 1.75 ^Aa^	33.31 ± 0.10 ^Ab^	94.11 ± 0.04 ^Ba^	41.15 ± 1.74 ^Ac^	76.28 ± 0.31 ^Bb^
Caffeoylquinic acid 2	<LOQ	<LOQ	<LOQ	<LOQ	<LOD	<LOQ
Cyanidin hex + Cyanidin deoxyhex-hex	nq	nq	nq	nq	nq	nq
Peonidin hex + Peonidin deoxyhex-hex	nq	nq	nq	nq	nq	nq
Caffeoylshikimic acid	<LOQ	<LOD	<LOD	<LOD	<LOD	<LOD
Quercetin pent-hex 1	3.36 ± 0.01 ^a^	2.50 ± 0.12 ^b^	2.36 ± 0.04 ^b^	<LOQ	<LOD	<LOQ
Quercetin deoxyhex-hex 1	12.79 ± 0.01 ^Aa^	<LOQ	8.27 ± 0.00 ^Ab^	8.66 ± 0.04 ^Ba^	<LOD	7.01 ± 0.05 ^Bb^
Quercetin hex 1	4.82 ± 0.04 ^a^	<LOQ	2.39 ± 0.14 ^b^	<LOQ	<LOD	<LOD
Quercetin pent-hex 2	29.25 ± 0.03 ^Aa^	19.36 ± 0.04 ^Ab^	19.38 ± 0.19 ^Ab^	5.78 ± 0.07 ^Ba^	2.86 ± 0.04 ^Bc^	5.17 ± 0.04 ^Bb^
Quercetin hex 2	7.52 ± 0.01 ^Aa^	2.70 ± 0.06 ^c^	4.57 ± 0.03 ^Ab^	3.88 ± 0.05 ^Ba^	<LOQ	3.13 ± 0.02 ^Bb^
Quercetin pent 1	5.08 ± 0.09 ^Aa^	<LOQ	2.90 ± 0.01 ^Ab^	2.69 ± 0.03 ^Ba^	<LOD	2.21 ± 0.02 ^Bb^
Quercetin pent 2	24.95 ± 0.14 ^Aa^	8.18 ± 0.05 ^c^	15.02 ± 0.07 ^Ab^	9.80 ± 0.09 ^Ba^	<LOQ	7.84 ± 0.06 ^Bb^
Methylquercetin deoxyhex-hex + Quercetin deoxyhex-hex 2	5.53 ± 0.10 ^Aa^	<LOQ	3.65 ± 0.02 ^Ab^	4.74 ± 0.04 ^Ba^	<LOD	3.63 ± 0.02 ^Ab^
**Quercetin pent 3**	54.56 ± 0.23 ^Aa^	19.77 ± 0.15 ^Ac^	32.85 ± 0.10 ^Ab^	25.47 ± 0.02 ^Ba^	6.43 ± 0.01 ^Bc^	22.11 ± 0.03 ^Bb^
Quercetin deoxyhex	10.76 ± 0.07 ^Aa^	6.55 ± 0.11 ^Ab^	5.91 ± 0.05 ^Bc^	6.54 ± 0.01 ^Ba^	2.84 ± 0.02 ^Bc^	6.27 ± 0.01 ^Ab^
Methylquercetin pent 1	4.65 ± 0.01 ^Aa^	<LOQ	2.71 ± 0.01 ^b^	2.47 ± 0.07 ^B^	<LOD	<LOQ
Methylquercetin pent 2	<LOQ	<LOD	<LOD	<LOD	<LOD	<LOD
Kaempferol deoxyhex	nq	nq	nq	nq	nq	nq
Methylquercetin pent 3	<LOQ	<LOQ	<LOQ	<LOQ	<LOD	<LOQ
Quercetin acetyl-(deoxyhex-hex)	<LOQ	<LOD	<LOQ	<LOQ	<LOD	<LOD
Methylquercetin acetylhex	<LOQ	<LOD	<LOD	<LOD	<LOD	<LOD
Quercetin	<LOQ	<LOD	<LOD	<LOD	<LOD	<LOD

PSE1—extracts of *P. spinosa* fruit from the location in western Serbia; PSE2—extracts of *P. spinosa* fruit from the location in central Serbia; Methanol—methanol extract of *P. spinosa* fruit; water—water extract of *P. spinosa* fruit; Ethanol 50%—hydroethanolic extract of *P. spinosa* fruit; hex—hexoside; deoxyhex—deoxyhexoside; pent—pentoside; nq—not quantitated; LOQ—limit of quantification; LOD—limit of detection. Hydroxycinnamic acid derivatives are expressed as chlorogenic acid (mg/100 g), flavonoids as quercetin (mg/100 g). The dominant compounds are marked in bold. Different letters ^(a–c)^ indicate significant differences (*p* < 0.05) between the extracts obtained with different solvents, ^(A,B)^ indicate significant differences (*p* < 0.05) between the extracts obtained from plants from different localities.

**Table 3:** Antioxidant activities and enzyme inhibitory effects (IC_50_) of blackthorn fruit methanol extracts.

	PSM1	PSM2	Positive Control (µg/mL)
DPPH (mM TE/100 g)	3.38 ± 0.10 ^A^	2.61 ± 0.17 ^B^	/ ^1^
ABTS (mM TE/100 g)	39.88 ± 0.12 ^A^	22.28 ± 0.37 ^B^	/
FRAP (mM TE/100 g)	2.51 ± 0.29 ^A^	1.57 ± 0.17 ^B^	/
*β*-carotene bleaching inhibition (%)	20.89 ± 2.28 ^A^	21.16 ± 2.43 ^A^	/
ACI (%)	90 ± 3.2 ^A^	67 ± 2.8 ^B^	/
*α*-Amy (mg/mL)	2.05 ± 0.05 ^A^	1.26 ± 0.04 ^B^	5.2 ± 0.16
*α*-Gls (mg/mL)	0.63 ± 0.02 ^A^	0.43 ± 0.06 ^B^	110 ± 10
AChE (mg/mL)	0.56 ± 0.08 ^A^	2.16 ± 0.25 ^B^	0.45 ± 0.06
TIA (mg/mL)	1.00 ± 0.07 ^A^	0.57 ± 0.02 ^B^	20 ± 0.2

PSM1—methanol extract of *P. spinosa* fruit from the location in western Serbia; PSM2—methanol extract of *P. spinosa* fruit from the location in central Serbia; ^1^ Trolox was used as a positive control to express activity in antioxidant tests; TE—Trolox equivalents; ACI—antioxidant Composite Index; α-Amy—α-amylase; α-Gls—α-glucosidase; AChE—acetylcholinesterase; TIA—tyrosinase; Positive controls: acarbose (α-Amy and α-Gls test), galantamine (AChE test), kojic acid (TIA test); Different letters within the same row indicate a significant difference (*p* < 0.05).

**Table 4:** Antimicrobial activity of blackthorn fruit methanol extracts.

Microorganism	Extracts		Antibacterial/Antifungal Agent			
	MIC (mg/mL)					
	PSM1	PSM2	AM	COL	CIP	FCZ
*Staphylococcus aureus*	5.0	5.0	4 (S)	N/A	0.001 (S)	N/A
*Staphylococcus epidermidis*	1.25	5.0	8 (S)	N/A	0.001 (S)	N/A
*Enterococcus faecalis*	>5.0	>5.0	N/A	N/A	2 (S)	N/A
*Bacillus subtilis*	>5.0	>5.0	N/A	N/A	N/A	N/A
*Escherichia coli*	2.5	5.0	8 (S)	0.5 (S)	0.125 (S)	N/A
*Klebsiella pneumoniae*	2.5	5.0	4 (S)	1 (S)	0.25 (S)	N/A
*Salmonella abony*	5.0	>5.0	4 (S)	0.5 (S)	0.125 (S)	N/A
*Pseudomonas aeruginosa*	>5.0	5.0	16 (S)	1 (S)	0.001 (S)	N/A
*Candida albicans*	>5.0	>5.0	N/A	N/A	N/A	2 (S)
*Lactobacillus plantarum*	>5.0	>5.0	8 (S) ^1^	N/A	N/A	N/A
*Limosilactobacillus reuteri*	>5.0	>5.0	16 (S)	N/A	N/A	N/A
*Lactobacillus rhamnosus* GG	>5.0	>5.0	4 (S) ^1^	N/A	N/A	N/A
*Streptococcus thermophilus*	>5.0	>5.0	0.125 ^2^	N/A	N/A	N/A
*Saccharomyces boulardii* 1	>5.0	>5.0	N/A	N/A	N/A	2 (S)
*Saccharomyces boulardii* 2	>5.0	>5.0	N/A	N/A	N/A	2 (S)
MIX 1 *L. helveticus*, *L. rhamnosus*, *B. longum*	>5.0	>5.0	N/A	N/A	N/A	N/A
MIX 2 *L. helveticus*, *L. rhamnosus*, *B. longum*, *S. boulardii*	>5.0	>5.0	N/A	N/A	N/A	N/A

MIC—minimal inhibitory concentration; PSM1—methanol extract of *P. spinosa* fruit from the location in western Serbia; PSM2—methanol extract of *P. spinosa* fruit from the location in central Serbia; AM—amikacin; COL—colistin; CIP—ciprofloxacin; FCZ—fluconazole; S—sensitive; N/A—not applicable; ^1^ Gentamicin was used instead of amikacin; ^2^ Erythromycin was used instead of amikacin. S-sensitive.

## Data Availability

Data is contained within the article and supplementary material.

## Supplementary Materials

The following supporting information can be downloaded at: https://www.mdpi.com/article/10.3390/foods11203289/s1, Figure S1: Blackthorn fruits, *Prunus spinosa* A—Crni vrh, central Serbia; B—Ljig, western Serbia; Table S1: Bacterial and yeast strains used in experiments; Table S2: Identified compounds in extracts of blackthorn fruits; Table S3: Correlations between total phenolic (TPC), total flavonoid (TFC) and total anthocyanin (TAC) content and antioxidant properties of blackthorn fruit extracts; Table S4: Prebiotic effect of blackthorn fruit methanol extracts; Figure S2: Stimulation of probiotic growth with blackthorn fruit methanol extracts.
