# Supplementary material for: Phenolic Composition, Antioxidant, Anti-Enzymatic, Antimicrobial and Prebiotic Properties of Prunus spinosa L. Fruits
Source: Foods. 2022 Oct 20;11(20):3289. doi: 10.3390/foods11203289 (PMC9602308; doi:10.3390/foods11203289)
Supplement: Supplementary file 1 [file foods-11-03289-s001.zip › foods-1975317-supplementary.pdf]

Supplementary Materials

A

B

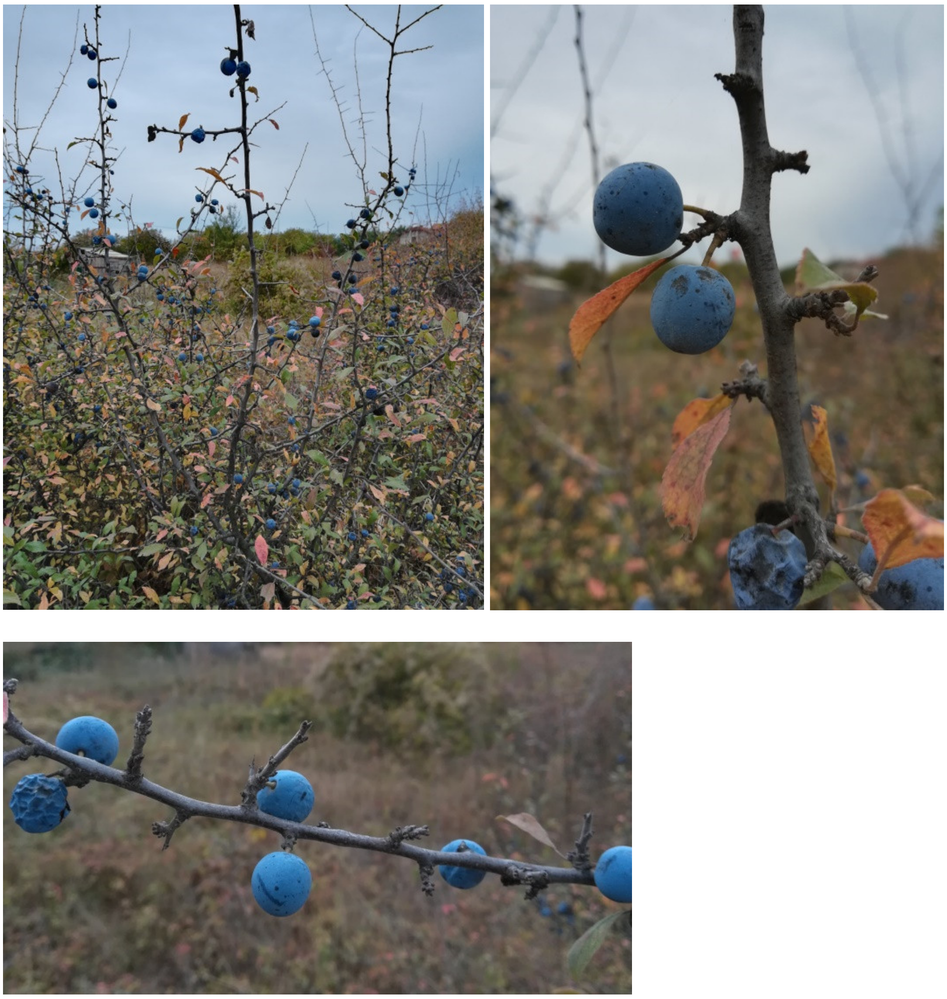

Figure S1. Blackthorn fruits, *Prunus spinosa* **A** - Crni vrh, central Serbia; **B** – Ljig, western Serbia

Table S1. Bacterial and yeast strains used in experiments.

**Pathogenic microorganisms**
**Gram-positive bacteria**
Staphylococcus aureus subsp. *aureus* Rosenbach ATCC 6538
Staphylococcus epidermidis ATCC 12228
Enterococcus faecalis ATCC 29212
Bacillus subtilis ATCC 6633
**Gram-negative bacteria**
Escherichia coli ATCC 25922
Klebsiella pneumoniae subsp. *pneumoniae* NCIMB 8267
Salmonella enterica subsp. *enterica* serovar Abony NCTC 6017
Pseudomonas aeruginosa ATCC 27853,
**Yeast**
Candida albicans ATCC 24433
**Probiotic strains**
**Gram-positive bacteria**
Lactobacillus plantarum Lp 299v
Limosilactobacillus reuteri Protectis (DSM 17938) (formerly *Lactobacillus reuteri*)
Lactobacillus rhamnosus GG (LGG)
Streptococcus salivarius subsp. *thermophilus* ST-21
**Yeast**
Saccharomyces boulardii strain 1
Saccharomyces boulardii strain 2
**Mixtures of probiotic strains**
**MIX 1** *Lactobacillus helveticus* Rosell-52, *L. rhamnosus* Rosell-11, *Bifidobacterium longum* Rosell-175
**MIX 2** *Lactobacillus helveticus* Rosell-52, *L. rhamnosus* Rosell-11, *Bifidobacterium longum* Rosell-175, *Saccharomyces boulardii*

Table S2. Identified compounds in blackthorn fruit extracts

t_R (min)	UV λ_{max} (nm)	ESI-MS data (*m*/*z*)	Assignment
10.1	254, 290	329, 167	Vanillic acid hexoside
11.1	236, 296sh, 324	353, 191, 179, 135	Caffeoylquinic acid 1
17.2	242, 296sh, 324	367, 193, 134	Feruloylquinic acid
17.8	240, 296sh, 328	353, 191, 179, 173, 135	Caffeoylquinic acid 2
21.0	280, 518	595, 449, 287	Cyanidin hexoside + cyanidin deoxyhexoside-hexoside
24.5	280, 518	609, 463, 301	Peonidin hexoside + peonidin deoxyhexoside-hexoside
26.6	234, 296sh, 326	335, 161, 133	Caffeoylshikimic acid
30.9	256, 266sh, 352	595, 300	Quercetin pentoside-hexoside 1
31.7	256, 266sh, 354	609, 300	Quercetin deoxyhexoside-hexoside 1
32.7	256, 266sh, 354	463, 300	Quercetin hexoside 1
32.9	256, 266sh, 352	595, 300	Quercetin pentoside-hexoside 2
33.2	256, 266sh, 354	463, 300	Quercetin hexoside 2
35.0	256, 266sh, 356	433, 300	Quercetin pentoside 1
35.8	256, 266sh, 356	433, 300	Quercetin pentoside 2
36.4	254, 266sh, 352	623, 609, 315, 300	Methylquercetin deoxyhexoside-hexoside + quercetin deoxyhexoside-hexoside 2
37.0	256, 266sh, 352	433, 300	Quercetin pentoside 3
37.4	256, 264sh, 350	447, 300	Quercetin deoxyhexoside
40.5	254, 266sh, 356	447, 314	Methylquercetin pentoside 1
41.3	254, 266sh, 356	447, 314	Methylquercetin pentoside 2
42.3	262, 344	431, 284	Kaempferol deoxyhexoside
42.7	254, 264sh, 354	447, 314	Methylquercetin pentoside 3
43.6	256, 266sh, 352	651, 301	Quercetin acetyl-(deoxyhexoside-hexoside)
44.4	256, 266sh, 354	519, 314	Methylquercetin acetylhexoside
46.0	256, 374	301	Quercetin

t_R – retention time. Mass spectra of phenolic acids and flavonoids were generated in the negative mode, whereas mass spectra of anthocyanins were acquired using positive mode.

Table S3. Correlations between total phenolic (TPC), total flavonoid (TFC) and total anthocyanin (TAC) content and antioxidant properties of blackthorn fruit extracts.

	DPPH	ABTS	FRAP	β-carotene bleaching inhibition	ACI
**PSM1**					
TPC	0.983^{***}	0.985^{***}	0.943^{***}	0.808^{***}	0.981^{***}
TFC	0.211	0.325	0.383	-0.287	0.138
TAC	0.722	0.449	0.643	0.962^{**}	0.773
**PSM2**					
TPC	0.942^{***}	0.333	0.766^{*}	0.924^{***}	0.928^{***}
TFC	-0.099	0.822^{**}	0.348	-0.336	0.123
TAC	0.897^{*}	-0.099	0.459	0.942^{**}	0.734

PSM1 – methanol extract of *P. spinosa* fruit from the location in western Serbia; PSM2 – methanol extract of *P. spinosa* fruit from the location in central Serbia; $^{*}p < 0.05$; $^{**}p < 0.01$; $^{***}p < 0.001$

Table S4. Prebiotic effect of blackthorn fruit methanol extracts.

			PSM1 (mg/mL)			
**Probiotic strain**	**5**	**2.5**	**1.25**	**0.625**	**0.313**	**0**
L. plantarum	132.5 ^{Aa}	124.0 ^{Aab}	117.1 ^{Aab}	104.8 ^{Ab}	105.5 ^{Ab}	100 ^{b}
L. reuteri	133.7 ^{Aa}	125.7 ^{Aab}	114.2 ^{Aab}	108.4 ^{Aab}	104.6 ^{Aab}	100 ^{b}
L. rhamnosus	127.2 ^{Aa}	117.0 ^{Aab}	113.4 ^{Aabc}	109.4 ^{Abc}	107.1 ^{Abc}	100 ^{c}
S. thermophilus	136.2 ^{Aa}	124.2 ^{Ab}	114.4 ^{Abc}	111.4 ^{Ac}	104.7 ^{Acd}	100 ^{d}
S. boulardii 1	139.7 ^{Aa}	126.4 ^{Aab}	117.3 ^{Abc}	111.2 ^{Abcd}	105.1 ^{Acd}	100 ^{d}
S. boulardii 2	148.9 ^{Aa}	143.9 ^{Aa}	129.8 ^{Ab}	111.1 ^{Ac}	110.5 ^{Ac}	100 ^{d}
L. helveticus, L. rhamnosus, B. longum (Mix 1)	127.5 ^{Aa}	120.9 ^{Aab}	111.8 ^{Abc}	110.0 ^{Bbcd}	108.1 ^{Acd}	100 ^{d}
L. helveticus, L. rhamnosus, B. longum, S. boulardii (Mix 2)	135.0 ^{Aa}	130.4 ^{Aab}	124.9 ^{Aab}	117.4 ^{Abc}	107.6 ^{Acd}	100 ^{d}
Mean ± SD	135.1 ± 7.0	126.6 ± 8.1	117.9 ± 6.3	110.5 ± 3.5	106.6 ± 2.1	100.0 ± 0.0
			**PSM2 (mg/mL)**			
**Probiotic strain**	**5**	**2.5**	**1.25**	**0.625**	**0.313**	**0**
L. plantarum	137.3 ^{Aa}	128.1 ^{Aab}	118.2 ^{Aabc}	110.6 ^{Abc}	104.8 ^{Abc}	100 ^{c}
L. reuteri	138.1 ^{Aa}	123.8 ^{Aab}	112.7 ^{Aab}	106.5 ^{Aab}	104.0 ^{Ab}	100 ^{b}
L. rhamnosus	136.6 ^{Aa}	129.0 ^{Aab}	121.9 ^{Aabc}	114.7 ^{Abcd}	110.7 ^{Acd}	100 ^{d}
S. thermophilus	140.3 ^{Aa}	128.5 ^{Ab}	114.4 ^{Ac}	110.4 ^{Acd}	99.3 ^{Ad}	100 ^{d}
S. boulardii 1	151.2 ^{Aa}	134.6 ^{Ab}	126.4 ^{Abc}	114.9 ^{Acd}	110.5 ^{Acd}	100 ^{d}
S. boulardii 2	152.8 ^{Aa}	145.2 ^{Aa}	129.8 ^{Ab}	118.4 ^{Ac}	111.5 ^{Ac}	100 ^{d}
L. helveticus, L. rhamnosus, B. longum (Mix 1)	141.8 ^{Aa}	129.4 ^{Ab}	122.7 ^{Abc}	114.4 ^{Acd}	109.8 ^{Ade}	100 ^{e}
L. helveticus, L. rhamnosus, B. longum, S. boulardii (Mix 2)	137.5 ^{Aa}	135.0 ^{Aa}	125.2 ^{Aab}	116.7 ^{Abc}	106.9 ^{Acd}	100 ^{d}
Mean ± SD	142.0 ± 6.4	131.7 ± 6.5	121.4 ± 5.9	113.3 ± 3.9	107.2 ±4.2	100.0 ± 0.0

PSM1 – methanol extract of *P. spinosa* fruit from the location in western Serbia; PSM2 – methanol extract of *P. spinosa* fruit from the location in central Serbia; Results are presented as percent of growth compared to the positive control of each probiotic strain (100 %). Different letters a–e indicate significant differences ($p < 0.05$) between the growths of probiotic species/strain treated with the increasing concentrations of same extract, whereas letters A–B indicate significant differences (p < 0.05) between the growths of probiotics species/strain treated with the same concentration of different extract (PSM1 and PSM2).

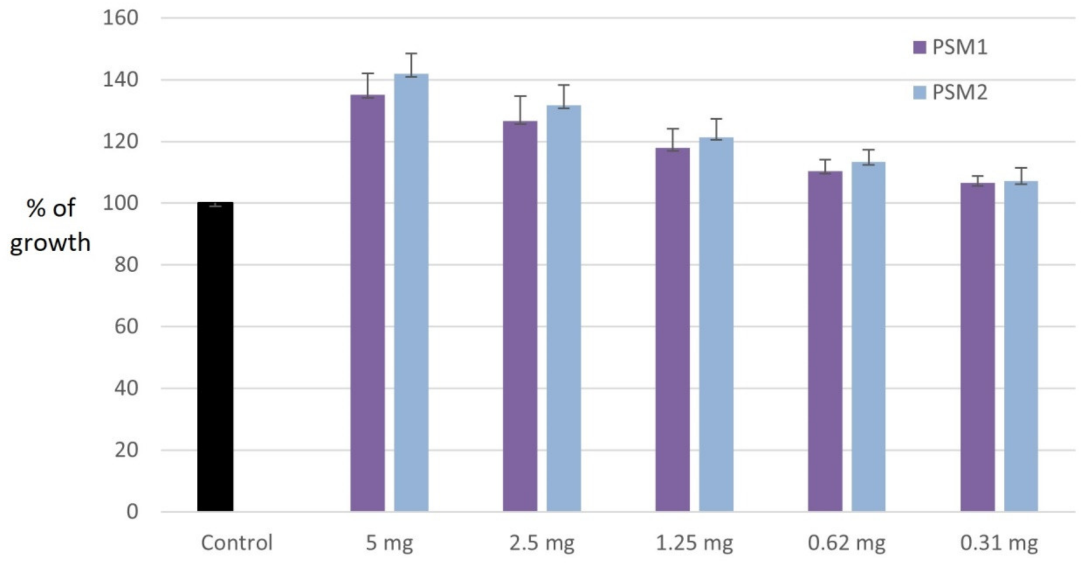

Figure S2. Stimulation of probiotic growth with blackthorn fruit methanol extracts.
PSM1 – methanol extract of *P. spinosa* fruit from the location in western Serbia; PSM2 – methanol extract of *P. spinosa* fruit from the location in central Serbia; Results are expressed as mean percent of growth stimulation ± SD of all tested probiotic strains (listed in Table S4) cultivated with PSM1 or PSM2 compared the growth of positive control (expressed as 100% of growth).
